# The Hospital Nursing Supplement

**Published:** 1893-09-16

**Authors:** 


### "The Hospital" Nursing Mirror.

Being the Extra Nursing Supplement of "The Hospital" Newspaper.

[Contributions for this Supplement should be addressed to the Editor, The Hospital, 428, Strand, London, W.C., and should have the word "Nursing" plainly written in left-hand top corner of the envelope.]

### News from the Nursing World.

### OUR NEEDLEWORK COMPETITION.

"When must our work be sent in?" is the query of various intending competitors, and to one and all our request is now made for all parcels to reach The Hospital office, 428, Strand, during the first week in December. They must be legibly addressed to The Editor, and bear in left hand corner the words "Needlework Competition." The name and address of the senders should be invariably enclosed in each package.

### INTELLIGENT PRECAUTIONS.

The word "cholera" appears just now in so many places in such large type that it seems a little incongruous to find the counter-balancing commonsense of the sanitary authorities is only set before the public in most unobtrusive small print. Even then it possesses the value of an antidote, and, if absorbed rapidly, it may render the poison of fright quite harmless. "And the moral of that is," if we may venture to quote "Alice in Wonderland" in so serious a relationship, that we had better reverse the order of things by taking the antidote before indulging in such deleterious nourishment as is embodied in sensational allusions to a cholera scare. By using our own commonsense and advising our neighbours to do the same, and to avoid shell-fish and over-ripe or under-ripe fruit, &c., we can face without fearing all reports of stray cases of true or spurious choleraic origin. If nurses continually strive to secure for themselves, as well as their patients, healthy minds in sound bodies, they will, indeed, be strong to defeat the attacks of the foreign enemy.

### AT LESS THAN COST PRICE.

Are bargains honest, and what are they? Professedly they are transactions in which the purchaser receives more value in kind than he gives back in coin. Why vendors should give their customers these advantages might puzzle a philosopher, but bargain-hunters are seldom given to logic, and the cheapness of an article apparently over-balances its claims to usefulness. Besides the regular contingent who scent from afar "things sold below cost price" (which is always problematical) there are sober-minded persons of both sexes to whom an occasional bargain offers the additional temptation of rarity. Some time ago the papers reported a case of a woman calling herself a nurse who was involved in certain transactions which included receiving money for goods which she undertook to obtain for something less than half the ordinary price. The lady who patronised and afterwards prosecuted her was, to say the least of it, thoughtless to imagine any dealer, whether wholesale or retail, was likely to make a stranger a present. So-called bargains should surely be viewed with distrust, lest "getting the best of it" in any transaction should prove little short of dishonest.

### MUCH TOO YOUNG.

Divers opinions as to the best age to begin training probationers have found their way into our columns. Some only can be considered instructive, but all are interesting as showing the various standpoints from which such a question should be viewed. The chief advocates of girl-nurses come from the ranks of youthful women who, having succeeded in gaining a certain amount of training in their teens, wish to have the same opportunities offered to all their friends. This desire is hardly likely to be carried out now that thoughtful attention is being paid to the welfare of both patients and probationers in all institutions where the sick poor are tended. The *British Medical Journal* said recently, in answer to a correspondent, "A young lady at the age of 20 is not, as a rule, either old enough or strong enough to enter upon the arduous work of training for the profession of a nurse in the wards of a general hospital." How much less suitable then that she should be admitted to the wards of infirmaries, where the work is harder and the cases more trying, or to a children's hospital, where the highest capacity as well as the most perfect health are essentials in the nursing of the helpless inmates.

### PEOPLE WHO "WANT TO KNOW."

There are persons who ask questions merely for their own pleasure. They seem to use them as substitutes for the conversational powers in which they are lacking. They cannot always wish for the knowledge which they ostensibly demand, because they often fail to heed the answers. There was once a young lady so addicted to interrogations that an irreverent boy cousin dubbed her "The Query," and this impromptu title became popular with her family. Foolish questions written or asked unfortunately tax the patience and take up the time of busy people. For example, a correspondent writes: "Is it customary or the correct thing to address nurses by their surnames?" and goes on to speak with indignation of the friends of a patient who had done so. Of course it is not customary, for if it were "the correct thing" it would not have caused surprise. Such bad taste is certainly exceptional, and probably few nurses have ever had to request their employers to use the customary prefix in addressing them. The simple word "Nurse" is generally found to satisfy both patient and worker, and the addition of the surname is quite unnecessary in private houses. Many sick persons never concern themselves at all with the proper or surnames of their attendants, which, after all, are matters of small importance, and only needed for subsequent reference.

### GRATEFUL NURSES.

In sickness nurses soon see the advantage of belonging to a hospital, for within its walls they secure the unwearied attendance of resident medical officers and nursing staff, the valuable visits of the consultants, and the companionship of fellow-workers. Nothing is grudged to the patients, who also find themselves the centre of much loving care. Whilst some may accept these attentions without much thought, others are gratefully appreciative of all that is done for them, and to the latter class belong four convalescent nurses who have recently sent us a pleasing letter telling of the good nursing and kind care which has surrounded them at the General Hospital, Nottingham. There was an outbreak of typhoid in the fever house, and two of these nurses were ill for five months. They have now been granted a long rest in which to recruit their strength, and the hospital authorities hope that they may be fit to resume their professional duties this autumn.

### AN IMPORTANT DECISION.

To secure good nurses for workhouse infirmaries it is obviously necessary to provide these institutions with trained Matrons and adequate Homes. The modern Nurses' Homes which already exist in connection with infirmaries are, as a rule, excellent ones, but the number of them is sadly limited. A practical object lesson on the difficulties of getting proper nurses where there is no proper Home was impressed upon the Leeds Guardians the other day, for after interviewing a number of applicants, most of those present agreed on the unsuitability of all of them for the post of assistant nurses which they aspired to fill. The need for housing and training probationers of a different class was admitted, and a long discussion followed as to the great expenses attendant on the erection of a Home. Common sense gained the day, however, and a motion was passed that the tenders, as recommended by the Infirmary Committee, should be accepted. Such an important town as Leeds ought certainly to be well to the fore in all wise progressive movements, and now that the Guardians have decided to build they are not likely to be satisfied until they possess a staff of certificated nurses and a training school for probationers which need fear comparison with no other in England.

### AT HOME AND ABROAD.

"Where can I find a doctor?" is a question which hardly needs answering in English cities. There are always several medical men living in each thoroughfare, and their whereabouts is easily discovered at a glance. When a consultant is desired in London, the visitor does well to make up his mind before entering a street in which each door-plate bears a name more or less famous, otherwise he will feel quite overwhelmed by the large choice which is offered to him. It is not so in Paris; the doctors are more distributed and less distinguishable. They have sets of apartments to which a concierge glibly directs the stranger, who eventually reaches a door which bears no indication of the owner's profession. On the other hand, the French medical man uses cards which set out in full his degree, and also his present connection with one or more hospitals, and the piece of pasteboard has a commercial look, which is absent from those bearing simply the name and address of the greatest English doctors.

### ENGLISH NURSES IN PARIS.

"There are plenty of English nurses already in Paris" is the discouraging reply received in answer to an inquiry made in that city as to the prospects for a new comer. Further questions elicit the information that there are certificated nurses, and some wearing "an order," presumably a badge, but the precise description of the latter is not forthcoming. Probably this is because the Parisian doctor as well as the practical Englishman sets no store by a decoration unless accompanied by satisfactory qualifications. English nurses are almost entirely employed by American or British visitors, and therefore the doctors have the responsibility of recommending them. The usual fee gained by nurses working on their own account is ten francs a day, but of course no one can get a succession of cases unless she is working for doctors who are able and willing to give her regular employment. It is therefore a first essential in setting up in Paris to know on what amount of support a nurse can depend, and unless she has sufficient encouragement she had better not face the risks attendant on making a start in a foreign country. To a qualified nurse, regularly employed by well-known doctors, private work in Paris has much to recommend it. The high fees facilitate living comfortably between cases in a furnished apartment, which, shared between two or three friends, is not costly. Then the numerous restaurants meet the necessity for proper food, and a spirit lamp provides the inevitable cup of tea at odd times. As regards the advantages of joining an institution, it is impossible to lay down a definite rule, but nurses should always remember that in making an agreement to work for an association of any kind, they are entitled to receive as well as to give "satisfactory references."

### GIFTS EASY TO CHOOSE.

Christmas seemed a long way off in recent days which overpowering heat have made memorable. But in spite of sun and cloudless skies the months pass swiftly on, and during the next three we want Christmas to be practically anticipated by our readers. We hope that many of them are going to send useful gifts for our annual distribution of clothing to sick adults who have to spend the great anniversary in hospital wards. We beg to remind our readers, young and old, whether they be under-worked or over-worked, whether they be rich or poor, that they can all do something to cheer the destitute and lonely. We hope that all those who can will take part in our needlework competition, but there must be also many workers who, not caring to try for a prize, could yet quite well make one simple useful gift. Presents such as these are easily contrived for those whose experience of Christmas Boxes is sadly limited, and who receive with pleased surprise those which reach them from kindly, unknown friends. It is difficult to select suitable offerings for the rich, but for the poor it is only needful to have something that is warm, useful, and, if possible, pleasant to the eyes. Therefore, we shall be glad to receive from our readers articles marked, "Not for competition," of the same description as those for which our prizes are offered.

### SHORT ITEMS.

The Nursing Sisters of the St. John Ambulance Brigade all hold both First Aid and Nursing Certificates. —The German railway companies set an example, which might well be followed elsewhere, in granting tickets at reduced fares to nurses going on holidays.

### Trained Nursing in Berlin.

*Prepared for the International Congress of Nurses.*

The Victoria House in Berlin for the nursing of the sick owes its origin to Her Majesty the Empress Frederick. Her Royal Highness desired to meet a two-fold need. First, she wished that the sick in their own homes, as well as those in hospitals, should have a share in the benefit of careful and humane nursing, afforded by good, trained, and educated women. Secondly, it was her Majesty's desire to open to young women of the educated ranks of life (but in poor circumstances and without occupation) an honourable, gratifying, and blessed vocation, free from all restrictions of the Roman Catholic confessional. Germany possesses, it is true, a great many evangelistic deaconesses and Roman Catholics, who do excellent work in the sphere of nursing, but on the other hand their number does not equal the demand, so that in a great many hospitals the nursing falls into the hands of uneducated and untrained male and female attendants, to whom the care of the sick is purely a means of livelihood. On the other hand, every woman is not willing to enter into a nunnery or a deaconess' home, although ready to assist her fellow men by devotion to a noble calling, and, in addition, to accomplish better and more satisfactory results in an organized and protected society than would be possible for one woman to achieve alone.

The Empress Frederick was anxious to gather suitable women into the society to which she had given her name, and to train them into thorough nurses. In the year 1881 her Royal Highness introduced her plan to the Society of Domestic Hygiene, of which she was the patroness.

The beginning was a small one. A house was dedicated and opened by her Majesty on January 4th. For the time being the superintendency was in the hands of a trained nurse of the Nightingale School in connection with St. Thomas's Hospital, London, who was assisted by three nurses trained in German hospitals, with the aid of the polytechnic society of the Board of Health. Nevertheless, a wider development was only possible by means of connection with public hospitals, and an opportunity soon presented itself; the Victoria House withdrew from the ranks of the Society of Domestic Hygiene, and three years afterwards, in the spring of 1886, it was organised as an independent society. Its labours proved so successful that at present, after ten years' independence, the Victoria House numbers 171 nurses who are actively employed in twenty-five different districts. This number does not include all the other graduates who, after having received their training and fulfilled their term of service, hold other positions as teachers in the art of nursing. The Victoria House is governed by a board of trustees consisting of ladies and gentlemen, of which the presiding chairman is the State Minister, Dr. Delbenck, who regulates the outside affairs of the Institution. A committee of four ladies and two gentlemen attend to the current affairs of the Home, the Chairman being her Excellency Frau Von Helenholz. Those accepted are young women and widows of the educated class between the ages of 20 to 35 years, with good character and sound health, without religious distinctions. Each candidate must produce a certificate of health, also a letter composed and written by herself, giving an account of her whole life; letters of recommendation from well-known persons, and in default of this a letter from her pastor, or a certificate of birth and baptism, and if possible a candidate should apply personally. Applications of those whom the principal finds acceptable are handed in by her to the chairman of the committee, and only those accepted by these two persons are received in the Victoria House. There is no stated time for entering; this depends entirely upon the number of deaconesses. After entering the Victoria House the probationer binds herself for three years—after receiving her training—to serve either in hospitals or in private houses, and to be subject to all rules of the institution. As a guarantee of the above obligation, she deposits in the bank of this institution 300 marks, which are kept for her and returned at 4 per cent. interest at the expiration of her agreement. In case of non-fulfilment of same, the money goes to the pension fund for nurses. The training of the nurses is carried on in one or more hospital, and requires one year; those less capable need longer time. Almost without exception the first six months are spent at the Universal Hospital at Fredricksheim, in Berlin, where an organised training school exists, and where the two chief physicians, Dr. Hahn and Dr. Faehringer, carry on the technical instruction which closes with a written examination. During the course of six months, they are all called pupil nurses, after that, "sisters." The Superintendent, who lives in the same house with the nurses, watches over them, and trains them for their future calling. In the first six months they are reserved for hospital service, and have a share in all the principal divisions of the work, being changed every three or four weeks to different posts. During the second half of the year they are considered as regular nurses, and have the opportunity given them of proving their fitness for bearing responsibilities. The Superintendent places them in different districts where they have practice and training in the care of the sick under the supervision of head nurses. At the end of the second six months, if they prove worthy, they assume the name and dress of "Victoria" nurses, and receive on November 21st, the Empress Frederick's birthday, also Commemoration Day, a silver Victoria medal, which is often presented by her Majesty's own hand; this medal they have to return in event of their leaving the Victoria Society.

During her training the Victoria nurse receives her board, lodging, laundry, and uniform free. At the end of the first half of the year she receives ten marks as pocket money. The Principal, with the Chairman of the Committee, is at liberty to dismiss at any time during her training, any nurse who does not prove worthy of receiving the training. The pupil nurses have a right to leave during the first three months by giving two weeks' notice. If one avails herself of this opportunity, or is dismissed on account of bad behaviour, she has to refund 50 marks per month as equivalent to her training. A rebate of 25 marks monthly is given if the cause of leaving is not the fault of the nurse. After admission as nurse at the Victoria House a certificate is given by the Superintendent and Committee which entitles to uniform, board, lodging, laundry, and a salary of 300 marks per year from the third year until it reaches 500 marks. In the case of those who show exceptional talent this salary is increased to 600 marks. With the exception of the abovenamed sum, the nurses are not allowed to accept any remuneration, and if any presents be given them they are to be handed over to the Institution. At the end of three years' service the Superintendent and Chairman have power to dismiss any of the nurses, but this only occurs in the case of serious grievances, and only then when repeated warnings have been unheeded. In the event of urgent circumstances, which makes the withdrawal of a nurse from the Institution compulsory, such cases receive prompt attention, and under these circumstances the Board of Trustees returns a nurse either in part or in whole the sum of money deposited by her. After the expiration of the three years a nurse can leave the Victoria House, after giving notice three months ahead, and on the first day of the month. In case of sickness the nurse receives free medical attention at the Victoria House or at any other hospital where she is working. Every year of service (excepting the first year) each sister is given a month's vacation, and is allowed reduction on the different railroads where she goes to take her holiday trip. The salary is continued during sickness and vacation. Sometimes at the end of her contract she receives as an extra compensation an extended period of vacation from three to six months, but during this time her salary ceases.

Each nurse is required to save 50 marks yearly to be put into the fund for aged and infirm nurses. This sum is deposited at the Prussian Renten Versicherungranstall. If a nurse is still in the service of the Victoria House at the age of sixty she receives a pension. If she leaves the institution before that age she receives all money she has deposited provided it has been in the Prussian Versicherungranstall five years, and after a year's notice is given she receives it back with compound interest. If a sister dies before the expiration of the above given notice, the money falls into the hands of the aforementioned bank. In addition to these amounts paid by each nurse, the Victoria House contributes 30 marks yearly to the same savings bank, which increases the pension for aged nurses. Should a nurse withdraw from the Victoria House before her sixtieth year then the contribution of the additional 30 marks yearly falls back to the Victoria House.

If a nurse becomes incapacitated before the age of sixty she is entitled to assistance from the Invalids' Fund of the Victoria House, which at present amounts to 56,713 marks. In each case the amount to be given is decided upon by the trustees. Besides these arrangements for disabled and aged nurses, there is another provision made for old age and accidents, although no nurse is entitled to its assistance until she is seventy years of age.

(*To be continued.*)

### The Position of Nurses.

By a Nurse.

The serious charges brought against nurses by different correspondents in The Hospital of August 19th seem to call for some reply. We are accused of attempting to supplant the general practitioner, of needing control from medical men, and of deterioration.

Having been a nurse for over fourteen years in London and Glasgow, and being now a jubilee probationer, I can perhaps explain some things which mislead the profession. Formerly, when poor people applied for a nurse, they were sometimes unable to get a doctor. Now they often grudge getting a doctor a second or third time, even if necessary, and a district nurse dare not put these cases off her books for fear the patient should be neglected altogether. The "overstepping" by the district nurse of the past was the outcome of sympathy with the poor, more than any intentional supplanting of the medical man.

The writer who complains of this supplanting does not call it right or wrong, but I say it is wrong, whether done by the district or specialist's nurse. I do not blame the nurses, but the medical men and the nursing schools. The medical men should lead the people, not follow them. What are we nurses but women of the period, plus helpmetes, as regards the medical profession, and no one can malign one without blaming the other? If doctors and nurses cannot settle the matter, I see no alternative but to put the whole profession under Government; but surely the discussion can be ended if we follow the plan of the Teachers' Guild. They have the teachers of both sexes in one guild, from the professor in the University to the humblest nursery governess, and everyone is free to speak his mind on any theme or lecture put before them, and if the ladies do not speak, they are accused of want of enthusiasm.

The spirit of competition is the master of the age, and co-operation is its servant. What is needed is, that doctors and nurses shall unite to reverse the situation, making co-operation the master, competition the assistant. If competition enters the sick-room, it is soon expelled by true men and women whose sole aim is the patient's recovery. The writer of the "supplanting" article says we nurses know "merely the rule of thumb" of our work and again, "nurses make themselves ridiculous by treading on the doctor's province, for they know nothing of it whatever." These ideas will need to be given up. They may apply to probationers but not to trained nurses, and no amount of sarcasm will ever make us accept inaccuracies. We are not nuns who have made a vow of obedience and buried our reason, and we carry out orders because it is our duty, and not because we are ignorant. At the Newcastle Congress, when a member asked for medical men to have entire control of every denomination of nurses, the doctors declined making the attempt.

Is this cowardice or is it wisdom on their part? Are they waiting, as medical men know how to wait, for a fit season? True, men and women know that the latter need controlling, especially the hard working, strong-minded ones who are apt, in their enthusiasm, to do too much. As every woman has a grain of sound influence in this world I will venture to give these lines, which I have copied :—

> " Some Water and Oil one day ha l a broil,
> As into a pot they were dropping,
> And would not unite, but continued to fight
> Without any prospect of stopping.
> So ne Potash overheard, and quick as the word
> It dropped in the midst of the clashing,
> When all three agreed uniting with speed,
> And Soap was invented for washing."

Being what your correspondent designates a "Common Woman," I feel able to undertake the defence, for I come from the lowest grade that could enter the nursing profession. I was educated in a simple way in a convent from the age of seven to twenty-one years, where I became a Government teacher, and when illness overtook me I afterwards got a post as sub-matron on a Government ship. We are accused of having the name of angel without deserving it, but we will keep it whether we deserve it or not; and all true, homely women share it with us whenever they nurse, whether as mothers, children's nurses, or sick nurses. The poet Scott wished us to climb, and we can do so by being humble, and instead of the notion that no one beats the modern nurse, let us remember there is always some distinct good in every race, and if the old nurses had not their food so well prepared, nor so many accomplishments as we have, they have left a kernel of good for us to evolve.

The Hospital writer would send us to convents, but having already been in one I have no intention of going back, for I do not like intellect to be cramped.

As for the uniforms, we will stick to them, no matter who imitates them, and we will offer something better for imitation with the uniform. As for nurses going out in white aprons without cloaks, it is quite startling to us Northern folk, and our surgeons would be down on us if we took the germs from promenades, theatres, &c., into their patients' rooms. Besides, you have to "behave yourself 'afore folk" in Scotland. With regard to remuneration, the writer says the pay of nurses is a miserable dole, and not calculated to encourage ladies to leave their homes. We have ladies in our midst whom it is a pleasure to meet, and they have benefited the profession, but they are women more for giving than for getting.

### Wants and Workers.

Can anyone give a nurse some information about women's work, including nursing, in California?—*L. M. N.*

### Nursing in South Africa.

BY A PRACTICAL NURSE.

### PRIVATE NURSING AND MISSION WORK.

A FEW days after my visit to the Somerset Hospital I had a letter from the Matron saying one of her nurses was down with typhoid, and would I take her place for three months? As I was expecting to move up country before the end of that time I offered to go for one month if I could be of any use, but the Matron wanted one nurse for the whole time. The same day I had a request from a lady to come to a case in her house, which it was feared might prove typhoid. I went, wondering much what my first experience of nursing in South Africa would be like; but never have I met with such kindness as in that household, nor been made to feel so completely one of the family. It turned out that the lady who was ill had a kind of malarial fever, which ran a curious course. Every alternate day the temperature rising to 103 deg., 104 deg. (and 105 deg. one night), commencing with a rigor; between times the temperature came down to 98·8, and the patient felt moderately well; this went on regularly for ten days. After four days of normal temperature she was allowed gradually to begin a light diet. There was diarrhœa on the eleventh day, but otherwise the motions were pretty regular and normal. In four more days she was allowed to get up for an hour or two in the afternoon, and after that the time was gradually extended, and she got back her strength very slowly. I was at the house three weeks, and the three children and I became great friends, taking many good scrambles together in daily walks up the hills, or along the streams caused by sudden heavy rains, or on the marshy flats. Nursingwas not my only occupation when indoors. The difficulty in getting servants here is very great, and one day we were left with only one young girl and a stop-gap for a cook, so we all had to help, the children making capital little housemaids, and having great fun over it.

To this lady I am indebted for the following facts about the nurses connected with the Cape General Mission, as she is on the Nursing Committee. This mission was started five years ago by the Rev. Spencer Walton, who, on a visit to the Cape, felt what a great need there was for something of the kind. It has grown most wonderfully, and there are branches in various parts of the colony. A nursing branch was started principally for district work. There is a Nursing Committee both in Cape Town and London; the hon. secretary in London is Miss Everett, 8, The Paragon, Streatham Hill, London. Anyone can apply to her for information, or to Mr. Mercer, 2, Berkeley Terrace, Wimbledon. Nurses are badly needed; they have to sign for four years, the passage money out is paid, and they receive £30 a year. The nursing embraces all kinds of district work, but the greatest need felt is for qualified monthly nurses and for certificated midwives. Sometimes the nurses are sent out to private cases; the extra fees paid for these go towards the maintenance of the Home, which is a comfortable old-fashioned Dutch house. Sister Janie is at the head, and she also goes out to poor women. It must be understood that this is real mission work, and only those are accepted by the English Council who feel called to devote their lives and their nursing powers as missionaries. Especially does the mission plead for self-supporting nurses. Many women with means come out ready to preach to the heathen, but so few trained lady nurses seem to feel the call. The population of Cape Town is a very mixed one—Malays, Kaffirs, Dutch, Indians, and a good proportion of English, so that the work is of necessity extremely interesting. The coloured people are so polite and respectful; I always receive a "Good morning, Missis," and a touch of the cap from the men, or a smile and a curtsey from the women and children whom I meet in the road. I hope these few words may stir up some nurses to come forward and give their services to this good mission work. There are seven nurses now, and for many more work could be found in this rapidly-increasing city of Cape Town.

### Every-Day Dangers.

### NURSING IN A COTTAGE HOSPITAL.

SOME interesting details concerning the nursing of the case of diphtheria at Watford District Cottage Hospital have been communicated to us by the late Sister Superintendent, Miss Catherine M. Hillyer. The fact of any person having to remain in constant attendance on a tracheotomy case for forty-three hours is well-nigh incomprehensible in a place near London, where the temporary aid of a trained nurse could be promptly secured. But it seems to have been the custom for the Sister Superintendent to nurse serious cases in the hospital by night as well as day, as she explains very clearly in her letter. It is certainly time that those responsible for the nursing of patients in cottage hospitals should realise that although patients may sometimes require equal attention by night and day, yet no one woman should be expected to do the work of two, however willing she be to attempt the double duty:

"In reading up the back numbers of THE HOSPITAL, I recently came across a paragraph (August 12th) headed 'Every Day Dangers,' which refers to a case of diphtheria at the Watford District Cottage Hospital. Will you allow me, in the interests of truth and of everybody concerned, to state the facts as they occurred? A child was sent into the hospital for the operation of tracheotomy, and I was not informed as to the nature of the case. Suspecting membranous croup, however, I at once put the child into the isolation ward, and took every precaution to prevent infection to myself or others. The case proved a fatal one, and owing to complications it was of a very distressing nature, requiring more than ordinary care and constant attention. I have seen a good deal of diphtheria, and have till this case nursed it with perfect impunity, both in and out of hospital, some being, of course, tracheotomy cases. Only a few weeks before I nursed two cases of scarlet fever in the hospital, and although I have never had the complaint myself, neither I nor any one else caught it. I entirely attribute my having in this instance fallen a victim to infection to the fact that for forty-three hours after the operation I was unable to leave my patient for a moment, consequently what food I had was eaten in the ward, proper rest and fresh air being, of course, out of the question. The hospital was nursed by myself and a nurse who had only had the training which the limited opportunities of a cottage hospital could give her, and she had never seen a case of tracheotomy. It would, therefore, have been culpable on my part to have allowed her to nurse it, even if she had not had to take charge of the other patients in the hospital. There had been an operation for strangulated hernia a few days before, which had entailed day and night work. I could not leave critical cases to untrained care, and was, therefore, sometimes obliged to nurse a patient day and night for a week together without any proper rest. This, of course, 'takes it out of one,' and a nurse is, I think, more liable to succumb to infection if she has been both mentally and bodily overtaxed, as I certainly was at the time. Most thoroughly do I endorse the concluding sentence of the paragraph already alluded to, that 'a nurse can no more hope to escape all contact with infection than a soldier can trust to his duty keeping him always out of gunshot range'; and I would carry the simile further, and add that no one expects a soldier to go into action single-handed, and if dangerous infectious diseases are admitted to a hospital there should surely be a sufficient staff to nurse them properly without unnecessary risk to any one person. The Committee presumably felt this when they censured the medical practitioner who sent in the case. Feeling that constant day and night work, such as I have had lately, was more than I could undertake in future, and being incapacitated for work by my illness, which lasted for some months, I sent in my resignation. The Committee, 'in reluctantly accepting' my resignation and that of my probationer nurse, expressed their sympathy with me, and made gratifying mention of my work at Watford, and presented us each with a sum of money "as a further testimonial of their appreciation of our services.'"

### Everybody's Opinion.

### A CORRECTION.

Miss Peter, Inspector Queen Victoria's Jubilee Institute, writes: Kindly allow me to correct statements made in an article in The Hospital of September 2nd, headed "The Parish Nurse," in Practical Information column. The Central Training Home of the Queen's Institute is 23, Bloomsbury Square. The nurses trained there are "Queen's Nurses," and supplied for town and country districts. One object of the Metropolitan and National Nursing Association is, as their latest report states, to train nurses for the Queen's Institute. Miss Oldham is Superintendent of the Rural branch of the Queen's Institute. Her address was formerly at 12, Buckingham Street, Strand, but now is Queen Victoria's Jubilee Institute, St. Katherine's, Regent's Park, N.W., where application for nurses for town or country should be made to the Inspector of Nurses.

### DISTRICT NURSING.

"A Nurse" writes: With all deference to Mrs. Dacre Craven, I do not think she exactly knows what district nursing is, or she never could have expected a nurse to do the work of a charwoman. The first part of her paper is all very well. Taking a temperature (if the doctor wishes it), making a patient clean, guarding against bed-sores, and if a surgical case, doing the dressings, and airing a room, but how can she expect a nurse to do all the other things she specifies if the nurse has a large and scattered district? I have been a district nurse for nine years', and until last November worked in four different parishes. (Last year my visits numbered 2,965.) The districts are now divided, and there are three nurses doing the work I did for eight years, single-handed. But if you were to ask each of those nurses, they would tell you they have as much as they can do of their own work (if they do it properly) without house work. I think it is time people should know that a trained nurse for the poor is not a "parish charwoman." Visitors amongst the poor often think a nurse should do everything in the house, and especially if the case happens to be a pet one. I have been told many times I ought to do this or that, but never by the poor themselves; they have always been grateful for anything done for them, and most kind and considerate. Again, if the nurse has the rough work to do, how can she keep her hands soft and nice to touch her patients? The poor seldom feel the contact of a soft hand, they are more used to the hard, though willing, touch of their own class. Many a time I have been asked to rub or put my hand on some one's head, "because yours is such a soft, light one." It may be a fad, but it is one of my fancies, that a nurse whether district or otherwise, should keep her hands as nice as possible. I once asked my committee how far I was authorized to do manual labour, and my answer was an emphatic "not at all." When they heard the facts of the case, they told me to get a woman to clean the place. I have not written this paper because I think it beneath me, or anyone else to scrub a floor (the work is sanctified, whatever it may be, if done in a right spirit), but simply to ask people to remember that if a nurs e is to do her own work properly, she cannot do other peoples. I am now obliged to give up my work on account of ill-health, but if I was strong and well I would again become (in spite of the hard work) what I have been for so long, a "District Nurse,"

### AN EXCELLENT DEFENCE.

"A Professional" writes: The time has now arrived for us to defend ourselves as much has been said and written lately about the members of the nursing profession by outsiders. Many nurses have not the time to do so, and many maintain a dignified silence, thus, perhaps, treating the matter with the contempt which it deserves. In our profession, as in every other, there are, I grieve to say, the bad and indifferent as well as the good, but the "Unprofessional Correspondent" has gone to the trouble to find out and state facts of the bad only. The bone of contention, the eyesore, grievance, call it what you will, seems to be the wearing of uniform by nurses, outdoor uniform in particular, and I do not see why a woman may not be allowed to claim her small privilege of clothing herself as she sees fit. Are we not distinct from other women? Soldiers and sailors are bound to wear the regulation dress, as a distinction from other men, and do we not do our duty as truly and honourably as they? why may not we wear a distinct dress without calling forth remarks from the outside world? A neat bonnet, with or without a veil, and finished off with neat white strings, and a cloak, will, ay, and does, command respect in town or country, travelling by rail or otherwise; and a woman thus garbed can, without fear, walk through the vilest slum at any hour of the day or night. The outdoor dress is only worn for a couple of hours a-day, on an average (except by district nurses), surely this is a small bait to tempt women to join the ranks of women who draw "high" salaries, and work twelve or more hours a-day. There are some women, no doubt, who answer to the description, so ably given in "The World of Nurses" (The Hospital, September 2nd), but surely these are in the minority. Consider the hundreds of women who day by day cheerfully fulfil unpleasant duties, and ask that the whole nursing body may not be judged by a few examples, thus "lowering the tone of the profession." All we ask of the public is to leave us alone.

### AN UNPROFESSIONAL CORRESPONDENT.

"An Old-fasioned Matron" writes: The note that "you cannot in any way be responsible for the opinions of your correspondents" is a most necessary one, and explains the appearance of the articles by the "Unprofessional Correspondent," who considers her or himself (I think the former) able to judge on a subject of which she has no knowledge—according to her own confession. She speaks as if self-sacrifice was not present, because a woman takes money for her work. Is not it self-sacrifice to work so hard, for such long hours, doing often such unpleasant duties (to call them by no harder name) as falls to the lot of all workers in hospital wards? Does the small stipend paid in cash represent an equivalent? I think not. I do not deny that motives, such as "catching a husband," may occasionally be an incentive for some light-headed girl to enter the nursing field, but as one, speaking with the authority and knowledge of twenty years of matronship, I say that such probationers are known to both matron and sister, and their speedy removal will probably follow, though your unprofessional correspondent will certainly not be informed. But dreadful as the sin may be the doctors will marry the nurses, and as I know well, find that they have chosen good wives. Surely (though I wander from my subject) artists marry artists, musicians marry musicians, actors marry actresses—but for a doctor and nurse to marry! according to your correspondent, ought to be a forbidden thing. I quite coincide with the strictures on the bad taste of nurses in caps and aprons at theatres—indeed I object to an out-door uniform, on principle, except for district nurses. But we all know (to our sorrow) that nursing uniform is worn by many as a disguise, and that the wearer has probably, except as a patient, never seen the interior of a ward. Matrons are to blame for allowing the style of hair dressing mentioned—it is a bad sign when a nurse frizzes her hair and mounts high heels. I fear our unprofessional correspondent is very much misinformed as to salaries, &c., and would advise the subject to be more closely inquired into. There is evidently confusion in her mind, and the perusal of some hospital reports would be beneficial to her. Your correspondent takes a different tone in her last article. Does she want the nurse to keep her salary in view all through her work, measuring what she does by her pay? Let anyone who has faithfully watched by a bad typhoid case, say whether it was the thought of pay-day, or the slight improvement, the fall of temperature, the quiet sleep at last, that filled her heart with joy as she wearily and lingeringly went off duty to well-earned rest. Does not a woman who has done this, thank God for letting her minister to His sick ones, and trust that faithful work for Him may be counted as self-sacrifice?

### Notes and Queries.

SPECIAL NOTICE.

The contents of the Editor's Letter-box have now reached such unwieldy proportions that it has become necessary to establish a hard and fast rule regarding Answers to Correspondents. In future, all questions requiring replies will continue to be answered in this column without any fee. If an answer is required by letter, a fee of half-a-crown must be enclosed with the note containing the enquiry. We are always pleased to help our numerous correspondents to the fullest extent, and we can trust them to sympathise in the overwhelming amount of writing which makes the new rules a necessity.

### Queries.

(190) *Registration.*—When are midwives to hold themselves in readiness for registration?—*Nurse P. S.*

(191) *Competition.*—Kindly tell me when articles should be sent in for needlework competition?—*Christmas.*

(192) *Cholera.*—Can you tell me of any published instructions on cholera nursing?—*Inquirer.*

(193) *Training.*—I wish to become a hospital nurse. Will someone kindly tell me how to set about it?—*Amy Bell.*

(194) *America.*—Please recommend a good American nurses' journal.—*I. McC.*

(195) *India.*—Where can I get information about Lady Roberts' Fund?—*J. P.*

### Answers.

(190) *Registration* (*Nurse P. S.*).—Midwives should qualify themselves for registration by passing the L.O.S. examination as soon as possible. When a Bill for the registration of midwives is passed the diploma of the London Obstetric Society will certainly be considered a registrable qualification. All information can be obtained by applicaiton to the Hon. Sec., Trained Nurses' Club, 12, Buckingham Street, Strand.

(191) *Competition* (*Christmas*).—They should be sent to The Hospital office, 428, Strand, in the first week of December, addressed legibly "Needlework."

(192) *Cholera* (*Inquirer*).—The recommendations of the council of the Q.V.T.I. are very good. We gave them in full in The Hospital of May 13th, page lxvii., in company with notes of Sir Joseph Fayrer's lecture on cholera, given to the Queen's Nurses. There is also a variety of information to be found in the following numbers of The Hospital, which can be obtained at the office, 428, Strand; September 3rd, 1892, page clxv.; September 17th, page clxxix.; December 17th, page xci.; and 1893, February 4th, page cxxxix.; and March 4th, page clxix.

(193) *Training* (*Amy Bell*).—Get "How to Become a Nurse," by Honnor Morten, published by Scientific Press, 428, Strand.

(194) *America* (*I. McC.*).—"The Trained Nurse," which is published monthly.

(195) *India* (*J.P.*).—In The Hospital of July 1st, page cxxxvi.

### Death in our Ranks.

Sister Flora Soward died at Margate on September 7th. Sister Soward had worked for the last two years for the Nursing Sisters' Institution, Devonshire Square, E., where she had endeared herself to superintendent, nursing sisters, and patients by her kind and unselfish disposition.

### For Reading to the Sick.

PRAYER.—III.

We might with advantage go through every petition of the Lord's prayer at length and see how beautifully it is suited to our needs, and how we can make it the model for every petition which we offer up to our Father which is in Heaven. To sum it up, however, in short, we ask that we may be daily conformed to the nature of our Heavenly Father, and that we may lead a heavenly life, keeping His Holy Name in reverence and honour, and yielding submissively to His will, however distasteful that will may at first appear, thus hastening the coming of God's Kingdom upon earth. Then having asked for grace for our souls, we pray that our bodily necessities may be supplied in the words, "Give us this day our daily bread," which covers everything that is needful for our food and sustenance. We say "bread," because it is commonly called "the staff of life," or the chief food support of our bodies, and it is also the type or figures of that life-giving bread, without which our souls cannot live, which came down from heaven to feed all Christians.

He gave Himself in either kind,
His precious flesh, His precious blood,
In love's own fulness thus designed,
Of the whole man to be the food.

Next we beg God to pardon all the evil we have done, and all the sins we have committed against Him, in the same way as we forgive those who have offended or injured us. Now this is a petition which requires very solemn thought, because it is, as it were, making a condition with the Almighty, and we should therefore be most careful to examine ourselves as to whether we do really show mercy to our fellow creatures. Perhaps, when we think about it, we shall say in our hearts, "No, I cannot quite forgive that unkind trick this person played on me, or the injury that others inflicted on those I love, so I will just leave those words out." But in acting thus we are making a vain attempt to deceive the Lord, who Himself framed this prayer for our use; we may not therefore omit any of it, nor try to shirk the duties it imposes. The Scripture says, "Forgive thy brother and thy neighbour the hurt that he hath done unto thee, so shall thy sins be forgiven thee when thou prayest." And another Scripture saith, "Blessed are the merciful, for they shall obtain mercy." We may take these words and ponder them well in our hearts, for, as we said before, all true devotion is founded on love, and it is only the love which thinketh no evil, but beareth all things and endureth all things, which can make us say properly "Forgive us, O Lord, as we forgive."

### Appointments.

[It is requested that successful candidates will send a copy of their applications and testimonials, with date of election, to The Editor, The Lodge, Porchester Square, W.]

Linacre Hospital for Infectious Diseases.—Mrs. A. Roberts has been appointed Nurse-Matron of the Linacre Hospital. Mrs. Roberts was Head Nurse at the Liverpool Eye and Ear Hospital, and afterwards had charge of four wards at the Bootle Borough Hospital. She takes many good wishes with her to her new work.

### Our Examinations.

Question for October.

What is hæmoptysis? What should be done when it occurs (1) in a hospital—(2) in a cottage?

Answers must be sent in by October 2nd. They must be clear and concise, written on one side of the paper only, accompanied by writer's name and address, and directed to "Nursing," Editor of The Hospital.

### The Nurses' Looking Glass.

### SOME THOUGHTS ON THE IMPORTANCE OF EARLY NURSERY TRAINING.

It has been said, "Heredity and environment are the master influences of the organic world. These have made all of us what we are. These forces are still ceaselessly playing upon all our lives. And he who truly understands these influences; he who has decided how much to allow to each; he who can regulate new forces as they arise, or adjust them to the old, so directing them as at one moment to make them co-operate, at another to counteract one another, understands the *rationale* of personal development. To seize continuously the opportunity of more and more perfect adjustment to better and higher conditions, to balance some inward evil with some purer influence acting from without, in a word to make our environment at the same time that it is making us, these are the secrets of a well-ordered and successful life."

There is no question that long before a child can speak intelligibly, it is taking in impressions and laying up for itself a store of observation against the future. It would seem, therefore, that one cannot begin too soon to regulate these impressions, and to surround the infant life with what is wholesome and right. You will often hear people say, "While baby is so young, it does not signify, &c.," but who is to decide how much baby, with its wide open, wondering eyes, is absorbing into its consciousness of the principles of baby life?

Long before it can tell us anything, or perhaps even itself distinguish the order of things, the outer life is beginning to mould the inner, and baby will, unconsciously to itself and to its relatives, develop later on in accordance with its nursery surroundings. These, if bad, may of course be corrected, it is to be hoped, in some measure later on; but we all know that work that has to be undone and done over again is never so good as that which is fresh and clean first hand.

To understand properly the needs and requirements of her children, every mother should have studied her own and their father's mental and physical characteristics, so as to know what faults and weaknesses, aye, and what virtues too, may have to be guarded against in the nursery discipline. For, be it remembered, the very virtues of manhood and womanhood are frequently the most obvious faults in childhood; in the self-reliance and independence of the man may be traced the conceit and self-will of the child, whilst the neatness and orderliness of the woman may in the child have taken the form of an undue care for externals, even to the extent of personal vanity.

It is scarcely doubted by most thinking people at this time that, as a rule, a mother is not the best nurse for her own children, even if she could (which is generally an impossibility), leave all other duties and relegate herself to the nursery entirely.

Children are the most exacting of tyrants, and they know almost before they can speak those over whom they can tyrannise. A tender, loving mother, with perhaps a sensitive nervous organisation, is a ready victim. She may, in brave defiance of her own feelings, be firm and wise in her control of her little one, but that little one knows all about it for all that, and will be twice as troublesome and unreasonable in consequence; hence the evil to the child. Its worst characteristics are brought to the front and intensified, instead of its best. We know a mother whose child's life almost depended upon her personal supervision, who yet was obliged to direct everything outside the nursery door, as the moment her face appeared inside complaint and whining took the place of smiles and contentment. The little one knew how closely the mother's eye watched over her, and in her baby tyranny loved to excite the anxiety, the agony of which she was happily unconscious.

The first responsibility, therefore, begins with the selection of a suitable nurse, and here a great mistake is often made by many who seem to imagine that the nurse so chosen is to remain, if she prove satisfactory, so long as the child requires one. Now there are very few people who are universal geniuses, and the servant class is no exception to the rule. A watchful motherly woman who might be invaluable to an infant, would probably be quite inadequate for the healthy development of a strong active child of four or five who wants a playfellow in the attendant quite as much as one who can wash and dress and protect from careless accidents. But in nine out of ten cases this fact is quite overlooked by the mother whose anxiety about the child is confined to a regard for its physical risks, and not its mental requirements. No one would imagine later on that the tutor or governess who was sufficient for reading and spelling and rudimentary Latin would be equally available to carry a pupil successfully through a competitive examination.

If the subject is looked at thoroughly, it will be seen that progressive teaching is the main element in all education, and ought to begin from the cradle.

In selecting for a child a thoroughly healthy, reliable, motherly young woman to take care of its infant life, there should always be the understood feeling that so soon as the child requires other and more active and intelligent companionship, this primary arrangement can be amended.

Of course, in many nurseries the annual advent of a new baby may keep the same nurse (if a good one) occupied for several years, but then she will, as time goes on, require help, and the help may take the form of the better playfellow for the growing little ones as they come on in succession.

Having now established as a fact that nursery changes are not only not an unmitigated evil, but often very real and substantial good to those most affected by them, we will only glance at one or two other influences which do not rank as important merely because people do not think about them, but which, nevertheless, it is possible may imperceptibly exercise a serious part in that moulding by environment which goes so far in making us what we are. First, there is the custom which exists in many families of sending the children at once on the arrival of visitors to be redressed, and probably sent down again with the injunction to "behave prettily to the company." How can it be wondered at if those children grow up with the idea that company dress and company manners are something quite distinct from what is necessary in everyday life; in fact, that anything will do for home, but the best things must be kept for society. The principle carried out in its entirety means a regard for appearances and a neglect of reality.

Again, a child told constantly, it may be only in fun, "Don't tell mother," will surely grow up with only a limited idea of the absolute trust which ought to characterise the holiest relation of life. The child, too, who is always brought up to expect a sugar plum as a reward for good conduct, will have a harder conflict when it comes to face the realities of life, and the sugar plums are found most frequently to drop into the mouths of the wrongdoers, than another who has been used at first only to obey because "mother says so," and who loves mother too well to doubt her wisdom, until as time goes on it can grasp the broader principle that "because right is right to follow right, were wisdom in the scorn of consequence."

Once more, the child who has been compensated for every trifling disappointment in the nursery will naturally enough look for similar results by-and-bye, and expect to find for every vexation some counterbalancing advantage; and when this fails, as fail it must, who shall estimate the bitterness of the disappointment resulting, may be, in damage to the moral tone, and an extinction of all healthy enjoyment of the ordinary pleasures of life.

To conclude, in what we have said our object has not been so much to lay down rules for other people's thought as to offer such suggestions as may help them to think for themselves, and by pointing out some of the pitfalls to which tiny lives are exposed, to establish a better understanding of baby's importance, and so to conduce to baby's good and ultimate well-being.

**THE BOOK WORLD FOR MEDICAL STUDENTS**—*continued.*

### BOOKS FOR SCOTCH STUDENTS.

[BY OUR EDINBURGH CORRESPONDENT.]

A considerable number of excellent medical books are issued by Edinburgh publishers, but for the most part they take the form of important monographs or elaborate treatises for the advanced student and the specialist, rather than of works suited to the wants of the ordinary student. Of these might be mentioned Byrom Bramwell's "Diseases of the Heart and Thoracic Aorta"; Bruce's "Mid and Hind Brain"; Webster's "Researches in Female Pelvic Anatomy"; Symington's "Topographical Anatomy of the Child"; and many others There are, however, a number of valuable text books and manuals which specially appeal to those preparing for examinations and to the junior practitioner. It is matter for Professor Patrick Geddes (second edition, 8vo., pp. 374, 408 illustrations, price 5s. Y. J. Pentland). It is a thoroughly reliable book, is written in a plain and simple style, and renders the study of botany most enjoyable. Mr. Alex. Johnstone's "Botany Notes" (pp. 116, price 2s. net, fourth edition. E. and S. Livingstone) are specially adapted for examination purposes.

ZOOLOGY.—Mr. J. Arthur Thomson's "Outlines of Zoology" (crown 8vo., pp. 642, 32 illustrations, price 12s. 6d. Pentland) is a most valuable work, the subject being treated in a thoroughly scientific, yet interesting, manner. For the student of medicine no better book can be had. "Illustrations of Zoology" (crown 4to, 400 figures, price 12s. 6d. Pentland), by W. R. Smith and J. S. Norwell, is an attempt to aid study by the use of numerous reliable diagrams, arranged in regret that in no one of the three great subjects into which medical science is divided—medicine, surgery, and midwifery is there a recent exhaustive systematic text book by any of the distinguished teachers of the Edinburgh School. The principles and practice taught in this, the greatest of our medical centres, have to be culled from magazine articles, hospital, and laboratory reports, and similar sources. Of less pretentious works there are several of great value in almost every subject, and in the special departments, such as ophthalmology, dermatology, and diseases of the ear, nose, and throat, are works which almost claim rank as classics. To refer to the subjects in the order of the curriculum: in

BOTANY.—Perhaps the best modern text-book is Dr. W. J. Behren's "Text-Book of General Botany," translated and revised by systematic order, the classification followed being that of F. M. Balfour. Johnstone's "Zoology Notes" is a companion volume to "Botany Notes."

Beyond small examination aid books, no student's work on chemistry comes from the Edinburgh Press.

ANATOMY.—A new edition of "Cunningham's Manual," in two volumes, is in the press. The second edition of J. Ryland Whitaker's "Anatomy of the Brain and Spinal Cord" (crown 8vo., pp. 166, 109 figs., many coloured; price 5s. net. Livingstone) is an invaluable work for the student of anatomy. It is fully up to date, and is beautifully illustrated. A somewhat lengthy appendix of tables and plates of nerves and blood vessels to Potter's "Compend of Anatomy" is of some value.

MATERIA MEDICA AND THERAPEUTICS.—Dr. W. Craig's "Manual" (8vo., pp 470; price 6s.; Livingstone) is in its fifth edition, which testifies as to its worth as a student's guide. Hare's "Practical Therapeutics" (8vo., pp. 624; price 16s.; Pentland) is a most satisfactory work.

PATHOLOGY.—Woodhead's "Practical Pathology" (third edition, 8vo., pp. 652, 195 coloured plates; price 25s. Pentland) is the standard work on the subject, and is too wellknown to require commendation. The improvements in the new edition place it far above all other books on practical pathology. Gibbes's "Practical Pathology and Morbid Histology" is illustrated with photographic reproductions which, however, are not to compare for teaching purposes with coloured drawing. Mr. T. Ryland Whitaker's "Notes on Pathology"

**THE BOOK WORLD FOR MEDICAL STUDENTS**—*continued.*

(pp. 245; price 4s. 6d. Livingstone) is intended to form a guide to the student in reading the standard text-books. It is deservedly popular.

Surgery.—The most recent, and one of the best text-books on surgery, is that edited by Keen and White. It is written by various American surgeons, and is fully up to date in pathology, and all modern methods of treatment. It is in two volumes, and costs 30s. net. (Pentland). Dr. Joseph Bell's "Manual of Operations of Surgery" (pp. 326, illustrated. Oliver and Boyd), a well-known work, is now in its seventh edition—a fact which speaks to its popularity. No student's library is complete which does not contain Caird and Cathcart's "Surgical Handbook," which is the most convenient, complete, and reliable guide to the practical parts of surgery we know. Wharton's "Minor Surgery and Bandaging" (post 8vo., pp. 498, 403 wood engravings, price 12s. 6d. Pentland.) is misnamed, inasmuch as it describes in detail all the ordinary amputations, excisions, and ligatures of blood-vessels, but it does it so well that it must rank as one of the best books of its class. The illustrations are excellent. Several works on Surgical Anatomy may be mentioned—*e.g.*, Shields', Hughes', and McLachan's—all equally good.

Medicine.—Undoubtedly amongst the best works on systematic medicine must be placed Osler's "Principles and Practice of Medicine" (large 8vo., pp. 1,080, charts, price 24s. Pentland), which embodies all the most advanced views on pathology, and describes the modern methods of clinical examination. It is thoroughly practical, and is written in a pleasing style. From the same press comes a "Compend of the Practice of Medicine," by Dr. D. E. Hughes, of Philadelphia, which is in its fourth edition. Gibson and Russell's "Physical Diagnosis" (second edition, crown 8vo., pp. 383, 109 illustration, price 10s. 6d. Pentland) is the standard work on the subject in Edinburgh. It reflects the teaching and methods of clinical investigation adopted in that school.

Midwifery.—Parvin's "Science and Art of Obstetrics," (2nd edition, large 8vo., pp. 701. 214 engravings. Price 18s. Pentland), and Winckel's "Text Book of Obstetrics" (royal 8vo., pp. 927, 190 engravings. Price 28s. Pentland), are both works which are modern, practical, and reliable.

Gynecology.—Perhaps the most practical, and at the same time the most scientific work on Gynecology in the English language is Hart and Barbour's "Manual of Gynecology." (4th edition, pp. 722, 414 illustrations. Price 18s. 9d. net. W. and A. K. Johnston.) The illustrations are numerous and instructive, and a copious bibliography is given. Dr. Halliday Croom's little "Manual of Minor Gynecological Operations and Appliances" is essentially practical.

No notice of Edinbnrgh published medical works for students would be complete without reference to "Pentland's Medical Series," of which three volumes have been issued. The first, "Diseases of the Skin," by W. Allan Jamieson (3rd edition, pp. 641. Price 16s. 8d.), has established itself as a medical classic, partly on account of the charm of its literary style, and also from the scientific manner in which the subject is considered, and the practical nature of its teaching.

George A. Berry's "Diseases of the Eye" is in its second edition (8vo. pp. 730, price 25s.), and is the most advanced text book available. The illustrations are coloured, and considerable space is devoted to operations.

Dr. P. McBride's work on "Diseases of the Throat, Nose, and Ear" (8vo., pp. 640, coloured illustrations, 25s.) is unique in embracing the three subjects in one volume. The writer states his opinions definitely, and leaves no ambiguity in the minds of his readers, a point of great value in a student's book. One has no hesitation in recommending the volumes of this series to students.

In a large school like Edinburgh it is impossible that all the medical works issued should reach the high standard of those mentioned above, and a crop of fungus literature has sprung up in the form of "Aids," "Catechisms," "Guides," and so on (for the most part unsigned), which are in no sense characteristic of the methods of the school. Their anonymous writers lack alike the spirit of the place and an appreciation of the best traditions of Edinburgh.

